# Vascular aging and cardiovascular disease: pathophysiology and measurement in the coronary arteries

**DOI:** 10.3389/fcvm.2023.1206156

**Published:** 2023-11-28

**Authors:** Daniel C. Y. Cheng, Rachel E. Climie, Matthew Shu, Stuart M. Grieve, Rebecca Kozor, Gemma A. Figtree

**Affiliations:** ^1^ Kolling Institute of Medical Research, Royal North Shore Hospital, Sydney, NSW, Australia; ^2^ Menzies Institute for Medical Research, University of Tasmania, Hobart, TAS, Australia; ^3^ Northern Clinical School, Faculty of Medicine and Health, The University of Sydney, Sydney, NSW, Australia; ^4^ Imaging and Phenotyping Laboratory, Charles Perkins Centre and Faculty of Medicine and Health, University of Sydney, Sydney, NSW, Australia; ^5^ Department of Cardiology, Royal North Shore Hospital, Sydney, NSW, Australia

**Keywords:** coronary artery disease, vascular aging, cardiovascular imaging, healthy vascular aging

## Abstract

Age is a key risk factor for cardiovascular disease, including atherosclerosis. However, pathophysiological disease processes in the arteries are not an inevitable feature of aging. Large cohort studies with arterial phenotyping along with clinical and demographic data are essential to better understand factors related to the susceptibility or resilience to age-related vascular pathophysiology in humans. This review explores the mechanisms by which vascular structure and function alters with age, and how these changes relate to cardiovascular pathophysiology and disease. Features of vascular aging in the coronary arteries have historically been difficult to quantify pre-mortem due to their size and location. However, non-invasive imaging modalities including CT Coronary Angiogram are now being used to assess coronary vascular age, and further advances in imaging analysis such as the CT Fat Attenuation Index will help provide further measurement of features associated with coronary vascular aging. Currently, markers of vascular aging are not used as therapeutic targets in routine clinical practice, but non-pharmacological interventions including aerobic exercise and low salt diet, as well as anti-hypertensives have been demonstrated to reduce arterial stiffness. Advances in imaging technology, both in acquisition and advanced analysis, as well as harmonisation of measurements for researchers across the globe will be invaluable in understanding what constitutes healthy vascular aging and in identifying features of vascular aging that are associated with coronary artery disease and its adverse outcomes. Assessing such images in large cohorts can facilitate improved definitions of resilient and susceptible phenotypes to vascular aging in the coronary arteries. This is a critical step in identifying further risk factors and biomarkers within these groups and driving forward the development of novel therapies aimed at slowing or stopping age-related vascular changes in the coronary arteries.

## 1. Introduction

### 1.1. Age-related cardiovascular risk

Age is widely considered a key risk factor for cardiovascular disease (CVD) (1). However, CVD is not an inevitable feature of aging as evidenced by “resilient” elderly with no disease (2). Historically it has been a truism that aging is synonymous with disease (3), with the contribution of age to cardiovascular risk variously attributed to the time-dependent nature of CVD processes, or to cumulative exposure to cardiovascular risk factors over time (4). In recent years, the paradigm has evolved to assign “age-related risk” to a complex interplay between the mechanistic and molecular effects of age on cardiovascular structure and function (4), and the specific pathophysiological mechanisms which produce disease (3).

### 1.2. Trends in aging and CVD

Coronary artery disease (CAD) constitutes a large proportion of overall morbidity and mortality attributable to CVD and is the leading cause of death worldwide, both in the western world and developing countries (5, 6). This continues to be the case, despite public health efforts targeting the prevention and management of CAD which have resulted in the age-adjusted death rate falling across the developed world (7). However, there has been an absolute increase in both the overall population incidence and prevalence of CAD due to increased longevity of both men and women and increased prevalence of traditional risk factors (8). In Australia, the shift towards an older population is expected to continue, with the over 65 year old population projected to increase by 57% from 2015 to 2030, compared to an increase in the general population of only 23% (9). A combination of these demographic trends and increased awareness of how age alters the structure and function of the vascular tree (so called vascular aging) has led to an increased focus on exploring the pathophysiology and management of this process (10).

### 1.3. Vascular aging

In the context of identifiable age-driven changes in the structure and function of the arteries, the term vascular aging has been coined (3, 11). More recently, there has also been a focus on defining and identifying patient groups who demonstrate increased susceptibility or resilience to the effects of aging on the vasculature (12–15) by classifying them into early and healthy vascular aging phenotypes. Defining these cohorts is critical in allowing further analysis of novel risk factors and therapeutic targets, as well as blood-based biomarkers which characterise these groups. However, there is currently no agreed definition of the parameters which characterise these phenotypes.

### 1.4. Vascular aging in the coronary arteries

The conceptualisation of the relationship between CAD and age has evolved to acknowledge the interaction of atherosclerotic plaque development with the pathophysiological features of vascular aging, which is also accelerated by traditional risk factors for atherosclerosis (16). From this perspective, key age-associated features including endothelial dysfunction, arterial stiffening, and intimal thickening serve as the foundation for the later development of atherosclerosis, with age also mediating the composition of plaque (17). Whilst these features are part of vascular aging, not all people who display them develop CAD. However, there have been limited attempts at identifying the conditions in which these features constitute physiological or healthy vascular aging, and when they lead to susceptibility to the development of CAD and related adverse events. Further characterisation of these factors may be possible utilising widely available imaging markers including coronary artery calcium (CAC) detected by Computed Tomography Coronary Angiogram (CTCA) (13), in conjunction with recently developed imaging analytics that can identify age-associated changes in the coronary vasculature (18). In this context, this review explores how age alters the structure and function of the vasculature, and our current understanding of how these changes are associated with the development of atherosclerosis. It then explores how the clinical manifestations of these changes are measured, with a focus specifically in the coronary arteries, the unique challenges that arise due to their size and location in the body, the current state of therapeutics in vascular aging, and recent advances allowing *in vivo* assessment.

## 2. Features of vascular aging—pathophysiology and relationship with coronary artery disease

**Figure 1:**
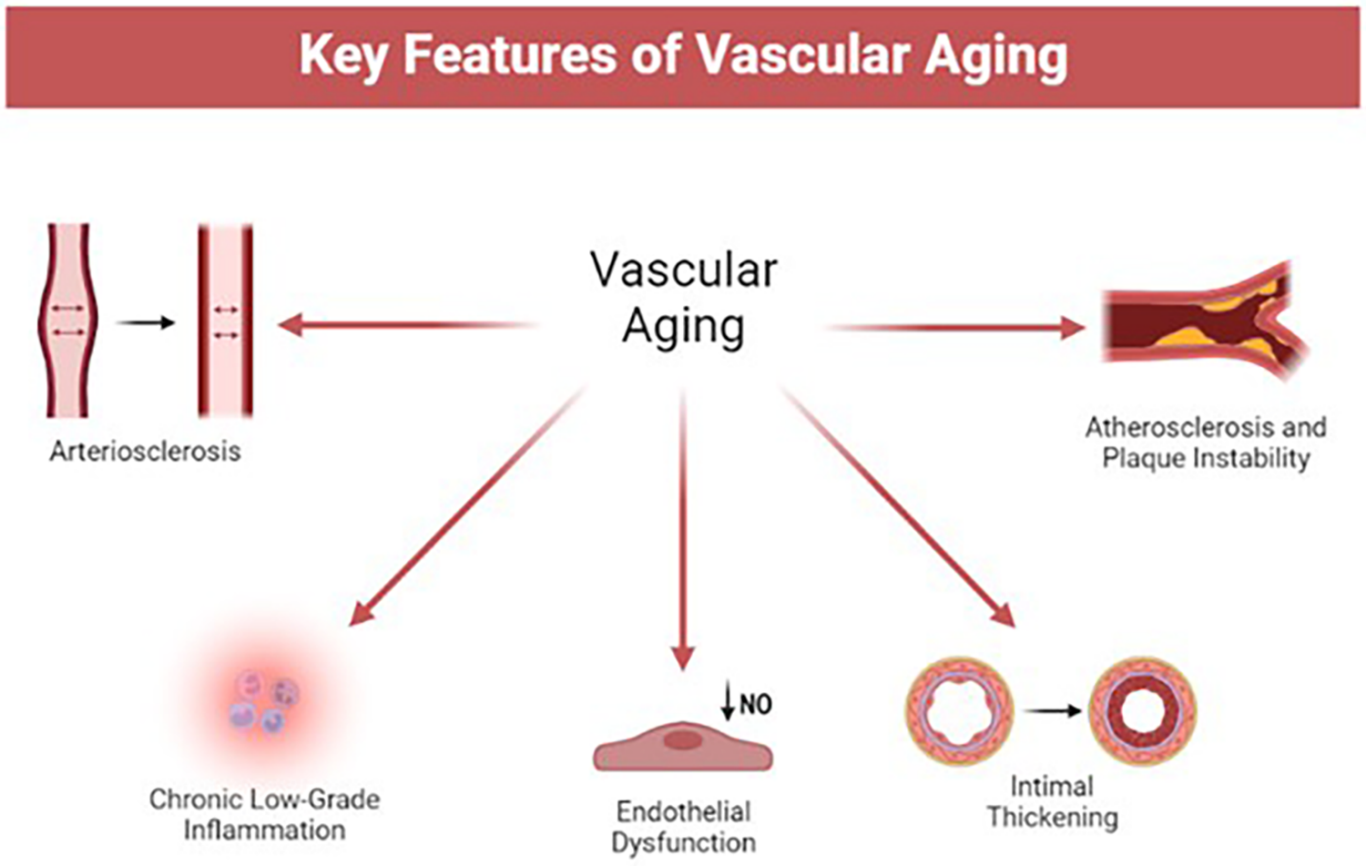
Features of vascular aging. NO = nitrous oxide.

**Figure 2:**
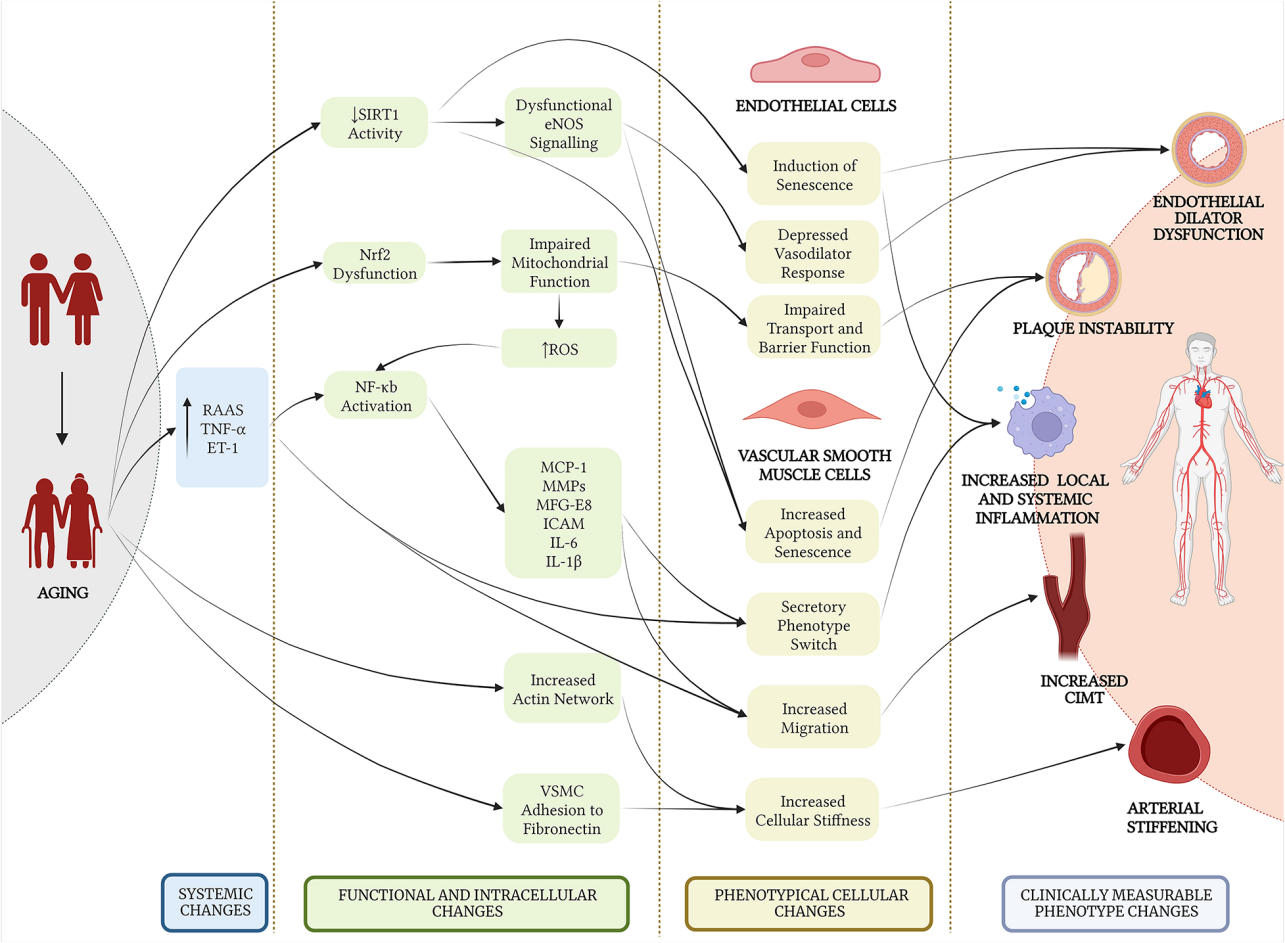
An outline of cellular changes of vascular aging. RAAS, renin-angiotensin-aldosterone system; TNF-α, tumor necrosis factor alpha; ET-1, enodthelin-1; eNOS, endothelial nitric oxide synthase; ROS, reactive oxygen species; MCP-1, monocyte chemoattractant protein-1; MMP, matrix metalloproteinase; MFG-E8, milk fat globule-EGF factor 8; ICAM, intracellular adhesion molecule; IL, interleukin; VSMC, vascular smooth muscle cell.

Figure 1 illustrates the known key contributors to vascular aging: (1) increased arterial stiffness, (2) intimal thickening, (3) chronic pro-inflammatory conditions, (4) endothelial dysfunction, (5) increased atherogenic conditions and formation of unstable plaques. Figure 2 outlines the cellular changes that drive these features of vascular aging.

### 2.1. Arterial stiffness

Arterial stiffness is a measure of the resistance of the arterial wall to dilation from an increase in volume within the artery (19) and is one of the key features of vascular aging. It is most commonly measured by pulse wave velocity in the large, elastic arteries such as the aorta, common femoral and carotid arteries. Whilst increased arterial stiffness occurs in a variety of disease states including hypertension and diabetes, it is most commonly found to be associated with aging (20). Aging has been demonstrated to lead to increased arterial stiffness by affecting both the endothelial cells and vascular smooth muscle cells (VSMCs) (21, 22) and by modulating the composition of the extra-cellular matrix (ECM) (23), all of which are key in maintaining arterial compliance.

Aging causes arterial stiffening by increasing matrix metalloproteinase (MMP) production and secretion from VSMCs (24–26) and also MMP activation (26, 27). This process is mediated by numerous pro-inflammatory cytokines. MMPs and cytokines play a key role in promoting vascular remodelling through collagenolysis and elastolysis (28), as well as collagen formation (29). These processes are thought to play a role in observed histological changes in large aging arteries, namely the increase in collagen and the collagen to elastin ratio (30, 31).

Aging has also been demonstrated to modulate cell-ECM interactions and VSMC stiffness, and there has been an increased focus on these mechanisms as a cause of arterial stiffening. Crosslink products of elastin including desmosine and isodesmosine have been shown to decrease in age, whilst pyridoline, the crosslink product of collagen has been demonstrated to increase (32). The combination of increased MMP activity, crosslink degradation, and repeated mechanical and oxidative stress on the arteries contributes to the rupture of elastic lamellae that has been observed with aging (33). Moreover, the increase in collagen and the collagen to elastin ratio in aging leads to more of the pulsatile force from systole being transmitted onto collagen fibres, which are 100–1,000 times stiffer than elastin (34), promoting further rupture of the elastic lamellae in the arterial wall and enhancing its rigidity. The changing composition of the ECM and resulting stiffness also drives cellular stiffness of VSMCs (22). Aged VSMCs have been demonstrated to exhibit cytoskeletal changes with a significantly more extensive actin network (20). They also show increased adhesion to fibronectin in the ECM, one of the key interactions that form the basis of the ECM-integrin-cytoskeletal axis that mediates the tone and compliance of the vascular wall to changes in volume and pressure (20).

The age-related cellular changes that lead to increased arterial stiffening are well described and clinical studies have demonstrated a positive association between arterial stiffness and coronary artery calcium (35). However, it is still unclear what drives the relationship between arterial stiffness and atherosclerosis (36). To further elucidate this relationship, identification of populations who exhibit early vascular aging as measured by arterial stiffening, and atherosclerosis will be helpful. Assessment of the identifiable changes in the ECM, VSMCs and endothelial cells, as well as any associated compensatory mechanisms in this group would provide insights into the mechanism through which arterial stiffness and atherosclerosis are linked.

### 2.2. Intimal-Media thickening

Subclinical changes in the structure of the arterial wall from the effects of aging start to appear from the second decade of life (37, 38). As we increase in age, the tunica intima starts to thicken as a physiological response to the forces acting on the vessel walls. This phenomenon is known as intimal thickening, and it has been observed that a two to threefold increase in the intimal-media layer occurs between the ages of 20 and 90 (39). The process occurs both in the large arteries and also within arterioles in the microvasculature (40). In fact, intimal thickening in the arterioles has a proportionally increased effect on lumen diameter and may adversely reduce end-organ perfusion (41). This thickening is made up of layers of VSMCs which have migrated from the tunica media and are intercalated between collagen and elastic fibres (37, 42). This process has been demonstrated to be driven by age related increases in angiotensin II and MGF-E8 signalling (43).

Intimal thickening manifests differently in the coronary arteries, where it occurs in an eccentric rather than concentric manner as in the aorta (44–46). The outer layer of the thickened intima has been demonstrated to have an enriched level of proteoglycans, and particularly biglycan, which has a high affinity for lipoprotein binding, a key process in early atherogenesis (47). Intimal thickening also leads to increased permeability of the vascular wall, which results in cholesterol and phospholipid deposition in the sub-endothelial space (48). This increase in permeability may increase susceptibility to hypercholesterolaemia and the development of atherosclerotic plaque, as has been demonstrated in mouse models (49, 50).

The degree of intimal-medial thickening in the carotid artery has been demonstrated to be predictive of future clinical cardiovascular events (51). However, carotid intimal-medial thickness has a strong, positive linear association with age even in healthy populations free from CVD risk factors (52). Thus, further exploration of when intimal thickening is a physiological part of the aging process and when it causes or is a marker of susceptibility to the development of atherosclerosis is needed, both in the coronary arteries and the wider vascular system.

### 2.3. Pro-inflammatory state

Vascular inflammation has been acknowledged as a key feature in atherogenesis (53), and there have been recent advancements in our capability to quantify this process in the coronary arteries by assessing change in the surrounding perivascular fat, as measured by computed tomography coronary angiogram (CTCA) and the fat attenuation index (FAI) (54). There is strong experimental and clinical evidence that chronic, sterile, low-grade inflammation in the vasculature is a key characteristic of aging, and plays a critical role in driving phenotypic shifts of both VSMCs and endothelial cells which further perpetuate a pro-inflammatory state (55). This multi-faceted process occurs via changes in key signalling cascades including increased renin-angiotensin-aldosterone system and endoethlin-1 receptor A activation and increased advanced glycation end products (55). This drives activation of pro-inflammatory signalling pathways including NF-κb, induction of pro-inflammatory cytokines including monocyte chemo-attractant protein-1, interleukin (IL)-6, IL-1β and tumor necrosis factor α, and increased oxidative stress and generation of reactive oxygen species (25, 56, 57). Downstream pro-inflammatory transcription factors in these pathways are upregulated whereas protective factors from inflammation have been demonstrated to decrease with age (56, 58–60). This pro-inflammatory environment drives vascular endothelial dysfunction and a pro-atherogenic environment through impairment of cellular metabolism, increasing oxidative stress and increasing apoptosis (61–64). The increased generation of reactive oxygen species has also been strongly implicated in driving microvascular dysfunction (65), which plays a driving role in age-associated end-organ damage (41).

The chronic low-grade inflammation in aging has been demonstrated to be a driving force behind a switch in VSMC phenotype. The environmental changes caused by aging include chronic inflammation, mechano-stimuli, cell death, calcification, and epigenetic events (66). These changes, especially the angiotensin II mediated chronic inflammation drive a modification in the VSMC phenotype from “contractile” to “secretory” (21, 43, 58). In their normal contractile state, VSMCs have a high expression of genes involved in the synthesis of proteins which are critical for myofilament structure and function, including *α*-smooth muscle actin, smooth muscle myosin heavy chain, and calponin (22). The secretory phenotype exhibits reduced expression of these genes as well as an increase in the production of pro-inflammatory cytokines, chemokines and adhesion molecules (22, 24). This pro-inflammatory secretory phenotype has also been demonstrated to be driven in part due to increased signalling of toll-like receptor 4 and Myd88 (67). This phenotype is also observed in intimal layer VSMCs, indicating that SMCs may dedifferentiate during migration from the medial layer to the intimal layer during the intimal thickening process which also occurs in aging (37).

Whilst the mechanism of inflammation driving endothelial dysfunction and the switch in VSMC phenotype has been described, it is unclear at what levels these changes mediate the development of CAD. With the advances and development of imaging resolution and analysis such as CT FAI, it is now possible to quantify localised levels of inflammation in the coronary arteries (18). This may enable us to define levels of localised inflammation that drive morphological and functional changes in the vasculature that lead to susceptibility to the development of atherosclerosis and CAD.

Aging has also been demonstrated to accelerate endothelial cell senescence, a process which is mediated amongst other factors by down-regulation of bradykinin type 2 receptor expression (68). One other pathway of growing interest that has also been demonstrated to mediate endothelial senescence is the age related reduction in activity of the encoding gene SIRT1 (69). This gene encodes for the sirtuin enzyme Sirt1, which is a NAD + (nicotinamide adenine dinucleotide) related histone-deactylase enzyme, and has been implicated in regulation of endothelial nitrous oxide synthase (eNOS) activity and expression, p53 acetylation and earlier induction of the stress-induced premature senescence phenotype in endothelial cells (70). Cellular senescence is part of the aging process, and is in essence an irreversible withdrawal from the cell cycle (71). Indeed, increased levels of endothelial senescence has been observed even in healthy, older adults (72), so it is yet to be identified at which point increased senescence drives susceptibility to disease. Evidence suggests that vascular endothelial senescence is inversely related with endothelial function, and thus contributes to the increasing endothelial dysfunction as we age (72–74). Endothelial senescence has also been linked to upregulation of production and release of a range of pro-inflammatory cytokines, contributing to an inflammatory environment which is a hallmark of the aging process (75). On top of this, the quantity and function of endothelial progenitor cells (EPCs) decreases as we age, and thus dysfunctional or senescent endothelial cells are not replaced at the same rate, which further perpetuates and promotes endothelial dysfunction in aging (76).

### 2.4. Endothelial dysfunction

The vascular endothelium plays a critical role in the regulation of vasculogenesis, angiogenesis, vascular tone and inflammation, as well as providing barrier functions for the vessel wall (77, 78). Multiple studies have indicated that aging contributes to endothelial dysfunction by increasing oxidative stress through the generation of reactive oxygen species by nicotinamide adenine dinucleotide phosphate oxidases, increased levels of arginase activity, and decreased eNOS activity (79–81). This age-related dysfunction is also mediated by gender, and has been demonstrated to occur ten years later in women compared to men (82). Crucially, this dysfunction depresses the endothelium-dependent dilator response by reducing the bioavailability of nitrous oxide in response to vasoconstriction and shear forces (83) and we are consequently able to measure the degree of endothelial dysfunction by assessing the vascular vasodilatory response to an external stimulus. Apart from being a critical vasodilator to help modulate arterial flow to match metabolic demand, nitrous oxide has also been demonstrated to have strong anti-inflammatory effects and inhibit leukocyte adhesion and VSMC proliferation (84–86). Resultingly, it has been demonstrated that sufficient bioavailability of nitrous oxide is critical to maintain arterial function (87). In the microvasculature, these alterations in endothelial function lead to impaired vasodilatory function of arterioles and higher capillary pressures which causes hyperfiltration, protein leakage and oedema (40). As the microvasculature is the key interface for delivery of essential oxygen and nutrients to the tissues, these age-associated changes are observed by assessing reduced flow or damage to end-organs including the brain, heart and kidneys (40).

Aging also affects endothelial function not only by impairing mechanotransduction of shear stress, but also by reducing laminar shear stress through increases in arterial diameter and reductions in blood velocity (88–91). Laminar shear stress has been demonstrated to maintain endothelial cell quiescence and function, stimulate eNOS expression, suppress endothelial proliferation and promote the expression of atheroprotective genes (92, 93). Disturbed and reduced shear stress accelerates endothelial proliferation and turnover, and promotes expression of atherogenic and thrombotic genes (94, 95).

Understanding how age drives endothelial dysfunction is critical in identifying the mechanisms through which populations with and without disease adjust to these changes. One study has demonstrated that healthy older males have higher levels of phosphorylated eNOS expression in endothelial cells compared to younger (96), which may be a compensatory mechanism in healthy vascular aging against reduced nitrous oxide bioavailability. Measurement of endothelial function in populations with CAD may enable identification and characterisation of the tissue level processes occurring in endothelial cells in both resilient and susceptible individuals. Further measurement of endothelial dysfunction in patients with clinical microvascular dysfunction but no epicardial coronary artery disease [commonly termed ischemia with no obstructive arteries (97)] may also shed light on what drives resilience and susceptibility to vascular aging in the microvasculature compared to the microvasculature.

### 2.5. Pro-atherogenic state and plaque instability

Atherosclerosis is a complex pathological process, mediated by numerous risk factors, including traditional ones such as hypertension, dyslipidaemia, smoking, diabetes and metabolic syndrome (98). Age-driven changes in the vasculature not only create a pro-atherogenic state as described above, but also influence the composition and vulnerability of the atherosclerotic plaque that is formed in the disease process.

Aged VSMCs have been shown to be more susceptible to apoptosis in mouse models, a phenomenon which has been demonstrated to be linked to dysfunctional eNOS signalling which occurs in age (99). VSMC apoptotic indices increase as atherosclerotic lesions develop, and chronic VSMC apoptosis which occurs in aging has been shown to stimulate plaque development and progression (100). Atherosclerotic plaque stability depends on the thickness of the fibrous cap and the degree of cap inflammation, with the most common precursor lesion to plaque rupture being thin-cap fibroatheroma (TCFA) (101). VSMC apoptosis in atherosclerotic plaques has been demonstrated to induce cap-thinning, breakdown of collagen and enlargement of the necrotic core, all of which increase plaque vulnerability (102). Aged VSMCs also display a reduction in proliferation rates and significant increase in the population doubling time, which is similar to VSMCs taken from advanced atherosclerotic plaques (21). They are also more likely to enter senescence or irreversible growth arrest, which is mediated by increased angiotensin II signalling seen in the chronic inflammation pathway associated with age (103, 104). In vitro findings also suggest that plaque stability and progression may be mediated by enhanced VSMC proliferation (21), as successful plaque repair is dependent on VSMC proliferation and synthesisation of ECM (105).

Impairment of mitochondrial function in the vasculature has also been demonstrated to result from aging, due to increased amounts of mitochondrial DNA (mtDNA) mutations (106, 107) and Nrf2 dysfunction which limits the efficiency of mtDNA repair mechanisms (25). The efficacy of the electron transport chain decreases with age, resulting in electron leakage and reduced levels of ATP synthesis (108). This dysfunction leads to increased production of reactive oxygen species, which once above a certain threshold continue to promote and perpetuate age associated damage of the mitochondria and surrounding vasculature (109). This dysfunction impairs the functional processes of the vessel wall including membrane transport and barrier functions, which are highly dependent on normal energy metabolism (25), and also promotes vulnerable plaque formation in mouse models (110–112). This was also demonstrated in a recent human study where it was shown that mtDNA damage was associated with the incidence of TCFA in the coronary vessels (113).

Whilst these genetic and cellular changes may suggest that TCFA prevalence should increase with age, a recent analysis of plaque characteristics showed that TCFA prevalence increases significantly with age in women but not men (114). This highlights a need to further explore the levels of VSMC apoptosis and mtDNA mutations in older women and men, and how they relate to the observed clinical manifestations. This will allow a better understanding of how these cellular and genetic changes drive susceptibility to vulnerable plaque development and the mediating factors of this relationship.

## 3. Clinical assessment of vascular aging

### 3.1. Arterial anatomy—regional differences

Arteries across the vascular tree are structurally composed of three different layers, the tunica intima, media and adventitia. The tunica intima is made up of a single layer of endothelial cells and a supporting layer of elastic tissue, on the luminal side of the artery wall. The tunica media is made up of elastic and muscular tissue comprised of elastin, collagen and VSMCs in varying amounts, depending on vessel size and location (24). The tunica adventitia consists of fibrous tissue and provides structural support and shape to the artery (115).

Whilst arteries across the vascular tree share common structural and cellular elements, there is significant heterogeneity in their composition in accordance with arterial location and function (116). For example, larger, elastic arteries such as the aorta and carotids have significantly more musculo-elastic complexes, with higher amounts of elastin in the ECM to help convert pulsatile pressure from the pumping of the heart into continuous laminar flow in the peripheral arterioles (24). The coronary arteries have lesser amounts of elastic tissue and higher numbers of VSMCs than the larger elastic arteries (117). The more distal peripheral arteries have the lowest amount of elastic tissue and highest numbers of VSMCs, as their primary function is to regulate vasomotor tone. These factors need to be considered when measuring changes between individuals, or in the same individual over time.

### 3.2. Measurement of vascular aging

The differences in the composition of arteries across the vascular tree result in variability in the way that they are affected by aging (11, 118, 119). This impacts how assessment of vascular aging can be performed, as not all the pathophysiological phenomenon that occur are easily measurable in the clinical setting. Moreover, not all parts of the vascular tree are easily measurable, especially the deeper, smaller calibre arteries. There has been limited exploration of the congruence in the assessed vascular age of participants between different investigations and the different locations of the vasculature they are measuring (120–122), and the findings that exist are inconsistent. One study demonstrated largely different results when measuring vascular age by carotid intima-media thickness (CIMT) compared to carotid-femoral pulse wave velocity (cfPWV) (121), and another showed a significant difference in the calculated vascular age when using CIMT compared to CAD burden reflected by CAC based calculations (122). On the other hand, one other study showed similarities between vascular age derived from CAC scores and CIMT (120).

Currently, clinical assessment of vascular aging has focused predominantly on assessment of arteriosclerosis as measured by cfPWV, which measures the stiffness of the descending and thoracic aorta (123). An overall vascular age can then be extrapolated from this regionally based measurement technique (124). The heterogeneity in the effects of aging on the vasculature underscores the importance of measuring vascular aging at the local level in territories such as the coronary arteries which are implicated in many of the adverse clinical outcome of CVD. This would better inform territory specific phenotypes of resilience and susceptibility to vascular aging for further investigation.

There has been limited exploration of how best to measure coronary artery aging using current imaging modalities, and very few assessments of the healthy or early aging phenotype in the coronary arteries specifically. Studies to date have primarily focused on atherosclerosis and coronary artery calcium as a measurement from which to derive vascular age, given atherosclerosis is the more commonly observed and easily measurable age-related pathophysiological change in the coronary vessels (13, 125). Moreover, there has been little to no exploration of how other characteristics of the vasculature that are associated with age including arteriosclerosis, intimal thickening, endothelial dysfunction, and low-grade inflammation, may be included in this assessment Table 1.

**Table 1:** Measurement of features of vascular aging- in health and disease.

Feature of vascular aging	Clinically measurable?	Clinical measurement method(s)	Measurable in the coronary arteries?	Measurement method in coronary arteries	Non-invasive?
Arterial stiffening	✓	▪ Ultrasound	✗	▪ N/A	▪ N/A
		▪ MRI			
Intimal thickening	✓	▪ Ultrasound	✗	▪ IVUS	✗
				▪ IV OCT	
Chronic low-grade inflammation	✓	▪ CT FAI	✓	▪ CT FAI	✓
		▪ High-sensitivity CRP		▪ PET	
Endothelial dysfunction	✓	▪ FMD	✓	▪ Quantitative coronary angiogram	✓
		▪ PAT			
		▪ EPC quantification		▪ IVUS	
		▪ MRI		▪ Transthoracic ultrasound	
		▪ PET		▪ MRI	
		▪ Quantitative coronary angiogram		▪ PET	
		▪ Intravascular ultrasound			
		▪ Transthoracic ultrasound			
Atherosclerosis and plaque instability	✓	▪ Ultrasound	✓	▪ CTCA	✓
		▪ CT		▪ MRI	
		▪ MRI		▪ IVUS	
		▪ Angiogram		▪ IV OCT	
				▪ Angiogram	

MRI, magnetic resonance imaging; IVUS, intravascular ultrasound; IV OCT, intravascular optical coherence tomography; CT FAI, computed tomography fat attenuation index; CRP, C-reactive protein; PET, positron emission tomography; FMD, flow mediated dilation; PAT, peripheral arterial tonometry; EPC, endothelial progenitor cell; CTCA, computed tomography coronary angiogram.

### 3.3. Measurement of arterial stiffening

Arterial stiffening can be measured at the systemic, regional and local level. Whilst systemic arterial stiffness can only be estimated from models of the body's circulation, regional stiffness can be measured via the assessment of pulse wave velocity between two arterial sites. Assessment of coronary arterial stiffening requires imaging techniques that can measure stiffening at the local level. Ultrasonography can measure the stiffness of the arterial wall by observing the change in pressure related to the change in volume within the artery (126–128). It can also measure the degree of intimal thickening within an artery, providing insights into the relationship between intimal thickening and stiffening (123). However, due to its high technical requirements and duration as an investigation, ultrasonography to measure local arterial stiffness is currently reserved for mechanistic analyses rather than epidemiologic studies (123, 126). Previous attempts to measure local coronary artery stiffness have mostly encountered methodological issues that preclude them from being used in broader clinical practice and research (129, 130). Moreover, measurement of the necessary parameters of pressure and velocity to assess stiffness in the coronary arteries can only currently be performed by invasive angiogram (130). In terms of non-invasive assessment, MRI measurement of local arterial stiffness is a novel field, with proprietary software enabling assessment for localised measurements of the aorta (131), but not yet the coronary arteries. Thus, there is limited scope to assess coronary arterial stiffness non-invasively with current imaging techniques.

Outside of the coronary arteries, measurement of arterial stiffness is the most common assessment used to define phenotypes of early or healthy vascular aging. Pulse wave velocity between the common carotid artery and the common femoral artery is the gold standard for measuring arterial stiffness, as it incorporates the aortic and aortoiliac pathways which are exposed to the greatest haemodynamic load from left ventricular systole (123), and thus are more significantly affected by arterial stiffening (126). Meta-analyses of large cohort studies including the Rotterdam and Framingham studies, have clearly demonstrated aortic stiffness as measured by cfPWV has an association with cardiovascular events and mortality, even once traditional risk factors are accounted for (132, 133). An analysis of 17,635 participants demonstrated a hazard ratio (HR) of 1.30 [95% confidence interval (CI): 1.18–1.43] for cardiovascular events and 1.23 (95% CI: 1.11–1.35) for coronary events per 1-SD change in log_e_aPWV (133) after adjustment for age, sex and traditional risk factors, and another of 15,877 participants demonstrated a pooled relative risk (RR) increase in cardiovascular mortality of 1.15 (95% CI: 1.09–1.21) for every 1 m/s increase in aortic PWV (132). It has also been demonstrated to be an independent predictor of cardiovascular outcomes in a range of populations (132, 134–136). There are multiple ways of measuring the pulse wave at the common carotid artery and common femoral artery, including transcutaneous pressure transducers (137), doppler ultrasound (138) and also MRI (131). This results in cfPWV being a simple, non-invasive, and reproducible investigation which makes it reasonably easy to use in the clinical setting, and as a means of assessing participants in research studies.

### 3.4. Measurement of intimal thickening

Measurement of intimal thickening is most commonly performed by assessing the carotid artery and has also been utilised in studies to assess the degree of vascular aging in different populations (139–141). However, there has been limited exploration of assessing intimal thickening in the coronary arteries, with invasive methods such as intravascular ultrasound (IVUS) or intravascular optical coherence tomography (IV OCT) used in specific small study populations including Kawasaki disease and cardiac transplant recipients (142–144). IVUS and OCT are both accurate measurements of intimal thickness when compared to histological measurement (142, 145). However, the invasiveness of these procedures limits their broader use in assessing subclinical levels of intimal thickening. High resolution transthoracic echocardiography has only thus far been able to detect intimal thickening in the left main and left anterior descending arteries (146, 147) and thus would provide an incomplete assessment of the major coronary arteries. MRI has been used to measure wall thickness in patients with established CAD in a few small study populations (148, 149). However, there are limitations to its broader use because of the higher technical and patient requirements, due to the smaller lumen and effect of both cardiac and respiratory motion on the vessels themselves (148).

Outside of the coronary arteries, carotid ultrasonography is non-invasive, safe and the most widely used clinical investigation which can measure the degree of intimal-media thickening of the common, internal and external carotid arteries. Whilst common femoral artery intimal-media thickening has also been demonstrated to have a positive correlation with CAD, it is less widely used in both the clinical setting and in assessments of vascular aging. Multiple meta-analyses of large cohort studies have demonstrated an association between CIMT and adverse cardiovascular outcomes including stroke and myocardial infarction (51, 150). Most recently, a meta-analysis of 119 randomised control trials (RCTs) demonstrated an association between intervention effects on CIMT progression and the risk reduction in cardiovascular outcomes [pooled RR of 0.91 per 10 µm/y reduction of CIMT progression (95% CI: 0.87–0.94)] (151), supporting its use in clinical trials as a surrogate marker of CVD risk. The additional predictive value of CIMT above and beyond the commonly used clinical risk scores is unclear, as multiple studies (150, 152, 153) have found that there was no significant improvement in these models with the addition of CIMT. Interestingly, in the Multi-ethnic Study of Atherosclerosis (MESA) cohort, addition of CIMT improved prediction from a baseline model of traditional risk factors included in the Framingham Risk Score for CAD events [Net Reclassification Index (NRI): 4.2%, *p* = 0.035 for presence of plaque] but not for the wider endpoint of CVD events, which included stroke (154). CIMT may be useful in identifying patients who are likely to have CAD and therefore an increased vascular age based on assessment of their coronary arteries, as a recent systematic review demonstrated that CIMT increased linearly with severity of CAD irrespective of its significance, and also showed a moderate correlation between carotid and coronary artery stenosis (155).

### 3.5. Measurement of inflammation

Age has been demonstrated to contribute to an inflammatory vascular environment through activation of a range of pro-inflammatory processes (156). There is also some evidence to suggest that aging may exacerbate the vascular inflammation caused by other cardiovascular risk factors such as obesity and hypertension, although this has not been well explored (157). Given the widely acknowledged importance of vascular inflammation as part of the process of atherogenesis, there have been concerted efforts to develop non-invasive localised measurement methods to improve cardiovascular risk stratification (54).

Recent developments in CTCA image analysis have led to the development of the FAI, which quantifies coronary vessel inflammation based on the change in attenuation of the surrounding perivascular adipose tissue (54). This has been shown through elegant bed to bench experiments, to closely reflect levels of inflammation and cytokine release from inflamed atherosclerotic plaque, and has additional prognostic utility (18, 158, 159). It has also been demonstrated to have prognostic utility in risk stratifying individuals with low or no coronary artery calcium based on the identified “residual inflammatory risk” as measured by the FAI (18). In the initial CRISP-CT derivation and validation cohorts, a high CT FAI value (defined as greater than or equal to 70.1 Hounsfield Units) was demonstrated to be predictive of all-cause [HR 2.55 (95% CI 1.65–3.92), *p* < 0.0001] and cardiac mortality [HR 9.04 (95% CI 3.35–24.40), *p* < 0.0001] (18) after adjustment for age, sex and traditional risk factors. These findings were supported in the SCOT-HEART trial, where further development of this analysis resulted in the creation of the fat radiomic profile (FRP) biomarker, which showed a signification association with major adverse cardiac events [HR 1.12 per 0.01 increase in FRP (95% CI 1.08–1.15), *p* < 0.001] (158). These results have also been supported by smaller *post-hoc* analysis of cohort studies in Asian populations (160, 161). However, at present, there has not been further validation of CT FAI's association with CVD outcomes in larger cohort studies, and it has not been incorporated or used in assessing vascular aging. In addition to FAI, FDG positron emission tomography (PET) has also been proposed as a non-invasive modality to measure low grade peri-coronary inflammation (162). However, given the recency of the interest in peri-coronary adipose tissue (PCAT), there have been limited studies exploring this imaging modality. One study found that PCAT uptake overall was higher in patients with CAD compared to non-CAD controls (163). It also found that FDG uptake in PCAT was positively related to the degree of stenosis in the respective coronary artery in patients with a body mass index greater than 25 (163).

Outside of the coronary arteries, vascular inflammation has been measured at the systemic level through high-sensitivity C-reactive protein. Measurement of this inflammatory marker has been demonstrated to predict future vascular events and improve global risk reclassification (164, 165), however it is poorly associated with local vascular biological processes of atherogenesis and inflammation (166). FDG PET has been utilised in studies to measure levels of vascular inflammation (167–169), and in assessment of patients with central and peripheral vasculitis (170). However, there are limitations to its wider utilisation for measurement of sub-clinical vascular inflammation. These include large variations in the imaging protocol and methodology between studies, the influence of patient factors including BMI and pre-scan glucose on results and the lack of validation in larger cohorts (169, 171, 172).

### 3.6. Measurement of endothelial dysfunction

Endothelial dysfunction has been demonstrated to contribute to the pro-inflammatory environment in aged vessels through the reduced bio-availability of nitrous oxide (84–86). The most direct and also invasive method used to measure endothelial dysfunction is intracoronary artery infusions, which involves using quantitative coronary angiograms or intravascular ultrasound to measure arterial diameter and blood flow velocity in response to a vasoactive intra-arterial infusion, most commonly acetylcholine (173). Acetylcholine is commonly used as it causes coronary artery dilatation in healthy endothelium, and a paradoxical constriction in dysfunctional endothelium (173). Whilst coronary endothelial function as measured by quantitative coronary angiogram has been posited as a superior risk stratification tool to the widely used Framingham Risk Score (174), it is not feasible as a screening tool or for epidemiological or large cohort studies due to its invasiveness and associated risk (175).

Another measure of coronary endothelial function is the coronary flow reserve (CFR), which is defined as the ratio between the maximal hyperaemic coronary blood flow over the resting baseline level (176). Fractional flow reserve, which is a ratio of the distal to proximal pressure in a coronary artery across an epicardial lesion during maximal pharmacological vasodilation, and CFR are being more commonly used to assess stenosis severity of CAD (177). However unlike fractional flow reserve, CFR can assess microvascular dysfunction independent of epicardial CAD (176, 178). Currently, CFR is most commonly measured invasively during angiography, and a CFR of less than 2.5 is diagnostic for coronary microvascular dysfunction (CMD) (97). Non-invasive measurement of CFR can be performed by transthoracic doppler echocardiogram assessment of the left anterior descending artery (179, 180), however this is limited as it is highly operator dependent due to the technical considerations of the scan and only measures one of the major coronary vessels. Two studies using pharmacologically induced stress echocardiography found an association between age and decreased CFR (181, 182). However, this has yet to be incorporated in any measure of coronary vascular aging. Other non-invasive measures of CMD include PET and cardiac MRI, through measurement of myocardial blood flow to calculate the myocardial perfusion reserve. This is a ratio of the maximum blood flow through the myocardium in response to stress compared to baseline (183). However, these non-invasive measures have been limited primarily to research rather than clinical practice given their high cost, limited availability, and long scanning time (184).

Higher field 3.0 T MRI imaging has recently been demonstrated in a few small studies to be able to measure endothelial-dependent coronary vasoreactivity to isometric hand grip exercises in both healthy and diseased patients (185, 186). However, to date this has been limited to studies with small sample sizes, most likely due to the difficulty from both a technical and economic perspective in ascertaining large groups of cardiac MRIs. Several studies have demonstrated that assessment of coronary vasodilator function with PET is an independent predictor of cardiac mortality in patients with known or suspected CAD (187–189). However, this marker is yet to be incorporated in any assessments of vascular aging.

Outside of the coronary arteries, endothelial dysfunction is measured based on the vasodilatory effect of nitrous oxide. This can be done through a variety of methods, with the most widely used and validated being flow-mediated dilatation (FMD) of the brachial artery (190). This is primarily due to the fact that it is non-invasive, easy to perform and efficient (190). A meta-analysis of 17,280 participants demonstrated a pooled adjusted RR for a 1% increase in FMD of 0.88 (95% CI 0.84–0.91) (191), demonstrating that FMD was a significant predictor of cardiovascular events in both population and cohort studies. This was reinforced by four other meta-analyses, which also used pooled effect models adjusted for confounding risk factors with similar results (192–195). However, some of these studies note that the methodological quality of the studies, and poor standardisation of measurement make it difficult to definitively assess its predictive capability beyond current traditional models (192, 193). Indeed, this was reflected in the MESA cohort, where the addition of FMD did not improve the Framingham Risk Score in terms of discrimination but did improve classification (NRI: 0.29, *p* < 0.001) (196). These factors have led to a position paper by the European Society of Cardiology which underlines FMD's function primarily as a valuable research tool (136). Other minimally-invasive methods of measuring endothelial dysfunction include peripheral arterial tonometry, and quantification of EPCs via an EPC-colony forming unit assay and measurement of circulating EPCs (190). However, both methods have significant drawbacks, as peripheral arterial tonometry measures micro vessel vasodilatory response as opposed to large vessels, and EPC assay results are highly operator dependent (190).

### 3.7. Measurement of atherosclerosis and vulnerable plaque

There is robust evidence that aging is a major risk factor for the development of atherosclerotic plaque and plaque vulnerability (197).

Measurement of coronary artery plaque severity and composition has been assessed invasively using coronary angiography, IVUS and IV OCT. There have been a few large cohort studies utilising IVUS to assess plaque characteristics associated with age. A study of 1,009 Korean patients identified severe calcifications and negative remodelling in more elderly patients with CAD, and more unstable plaque morphology in younger patients (198). Another study of 990 patients demonstrated an association between age and the percentage of the calcium and lipid components of plaque (199). A third study of 697 reinforced these findings and also found higher rates of TCFA in patients over the age of 65 (200). IVUS has also been utilised in multiple RCTs of hundreds of patients investigating the impact of statin therapy on coronary atherosclerosis burden (201–203). IV OCT has been demonstrated to be superior than IVUS in quantifying coronary calcium and characteristics including thickness, area and volume due to its greater penetration (204). However, all these methods are difficult to use in population level community-based assessments of vascular age, given the invasive nature of the procedures and associated risks.

The most common non-invasive method of assessing coronary artery pathophysiology associated with aging is the measurement of coronary artery calcium deposition using a harmonised coronary artery calcium score. This is a standardised, non-invasive and low radiation study without contrast, providing a score reflecting coronary atherosclerotic burden that is strongly prognostic of future cardiovascular and coronary artery related events (205). CAC scoring using CTCA is widely available and relatively cost-effective in patients with suspected atherosclerosis (206–208). It also has the major advantages of being non-invasive, and able to visualise the vessel wall and high-risk positive remodelling rather than just luminal stenosis as may be assessed by invasive coronary angiography. The latter is particularly important when defining “healthy” aging, as diffuse non-obstructive CAD may not be well-appreciated by traditional invasive angiography (209). CAC scoring has been shown across multiple cohort studies including the Rotterdam Study [HR: 2.4 (95% CI 1.3–4.5)] for CAC 401–1,000 compared to CAC 0–100, MESA [HR: 3.61 (95% CI 1.96–6.65) for doubling of the CAC score], and the CAC consortium [HR: 1.45 (95% CI: 1.15–1.83) for CAC 1–100 vs. CAC 0] to be associated with an increased risk of adverse cardiovascular events (210–214), irrespective of ethnicity and independent of age, sex and traditional risk factors. This association was initially confirmed in middle-aged and older adults, and is also supported in adults as young as 30 years old by a significant association between CAC score and CVD mortality [HR for CAC > 100 vs. CAC 0: 3.3 (95% CI 1.8–6.2)] (215). CAC has also been demonstrated to add predictive value above traditional risk factors in the MESA [NRI: 0.25 (95% CI 0.16–0.34, *p* < 0.001)] and Heinz Nixdorf Recall (NRI: 0.224, *p* = 0.0009) studies (205, 207). Given this strong association with outcomes, randomised screening trials are now looking at the viability of using CAC to risk stratify patients for adverse coronary events instead of traditional risk scores, however the longer term outcome data is yet to be published (216, 217). Advances in high resolution CT have enabled measurement of coronary artery lumen and vessel wall dimensions as well as characterization of plaque composition (218, 219). This may enable more precise identification of patient phenotypes that are resilient or susceptible to the pathophysiological effects of aging on the vasculature through identification and quantification of vulnerable plaque and TCFA (207).

### 3.8. Risk factors of vascular aging

In the context of the heterogeneous speed of vascular aging across the population (220), it is important to understand the risk factors of vascular aging which may help in the identification of phenotypic groups. Non-modifiable risk factors that have been identified outside of chronological age include sex, ethnicity, family history, genetic factors and prenatal fetal growth (15, 221). Younger women have been observed to have slower rates of vascular aging, however, experience an increase in the rate of vascular aging in their 60’s as opposed to in the 70’s for men (220). This earlier increase has been thought to be associated with the onset of menopause (222). The sex differences in vascular aging have been shown to differ across vascular beds, highlighting the need for clearly defined measurement targets and assessment tools for localized vascular aging, including in the coronary arteries (222).

There are a wide range of modifiable risk factors that have been associated with early vascular aging including traditional cardiovascular risk factors (223–227), and there is also emerging evidence of association with depression in men and individual deprivation (228, 229). Individual risk factors do not necessarily affect all of the pathophysiological manifestations of vascular aging homogeneously, as the CRAVE study demonstrated that patients with hypertension and dyslipidemia had a four times higher rate of progression of cfPWV than those without, but nil effect on endothelial dysfunction as measured by FMD (230). Longitudinal data has also demonstrated the impact of early life pathology and even fetal stressors on vascular aging (231), and how ongoing exposure to risk factors can mediate its trajectory. In one study, metabolic syndrome in children and adolescents predicted arterial stiffness in middle aged adults (232). Another recent longitudinal study examining the trajectories of vascular aging as measured by a range of functional and structural indicators including CMIT and cfPWV found that lifestyle risk factors including smoking, diet, step count, BMI and HbA1c were associated with the trajectory of vascular aging over 11 years (220). They also reported a significant association between grip and leg strength and an increase in CIMT, and trunk flexibility and decreased cfPWV (220), which is consistent with prior studies (233, 234). Habitual aerobic exercise has also been demonstrated to mitigate the decline in endothelial function with age (235), however the effect of resistance or anaerobic training is more unclear.

Defining risk factors that are associated with coronary arterial aging is difficult with the currently available non-invasive assessment options, as it is difficult to delineate risk factors of atherosclerosis as an independent disease entity or as a marker of vascular aging. The heterogeneous effect of different risk factors on the clinically measurable manifestations of vascular aging highlight how characterization of indices of localized vascular aging in the coronaries outside of atherosclerosis is important in further understanding the disease process. Thus, incorporating novel, widely available, non-invasive measures such as CT FAI is integral in being able to perform assessments of the impact of risk factors of coronary arterial aging.

## 4. Measurement of coronary arterial age

Studies have used CTCA imaging data in different ways to assess the vascular age of the coronary arteries (121). The continuing absence of CAC has been used to define a healthy vascular aging phenotype in the Multi-Ethnic Study of Atherosclerosis (13) and Bogalusa Heart Study (12). McClelland et al. proposed a method of transforming the measurement of CAC from Agaston units into an arterial age, which they defined as the age at which the estimated coronary heart disease risk is the same for the observed CAC score (236). They demonstrated that using this CAC-derived arterial age instead of observed age resulted in a Framingham Risk Score that was more predictive of short-term incident coronary events based on data from the Multi-Ethnic Study of Atherosclerosis (236). Further studies using this calculation have also demonstrated that adding CAC-derived arterial age to a predictive model for stress-induced myocardial ischemia based on clinical data resulted in a higher net benefit than using chronological age (237). Another study however has suggested using an age adjusted segment involvement score as a surrogate marker of vascular age, which was demonstrated to have an incremental prognostic value to traditional risk factor evaluation based on data from the Coronary CT Angiography Evaluation for Clinical Outcomes: An International Multicentre study (238).

Expanding the assessment of coronary arterial age beyond atherosclerosis by incorporating other features of vascular aging is becoming possible with advances in imaging resolution and analysis. Whilst IVUS has been used to identify specific age-associated plaque morphology, these are yet to be incorporated into an assessment of coronary arterial age, given the necessity for an invasive procedure. CT FAI has made possible assessment of inflammation in the coronary arteries, however a standardised system of scoring has only recently been developed. Initial cohort studies of this scoring system demonstrate an increase in the FAI-score with age (239), as would be expected with the inflammation associated with vascular aging. Advanced imaging analysis (240) can also allow us to identify salient features on CTCA that are associated with aging and explore any prognostic significance they may have in being incorporated into an assessment of coronary arterial age.

## 5. Therapeutics of vascular aging

Currently available pharmacological therapies for vascular aging focus primarily on the effect on pulse wave velocity, as it the most common way vascular aging is measured and defined (15). Some studies have also assessed response via measurement of endothelial function through FMD (241, 242). However, there is limited evidence for using a reduction in cfPWV as a treatment target in hypertensive or diabetic populations, and no evidence to support therapies in non-hypertensive or non-diabetic populations (15). Whilst many assessments of pharmacological therapies have assessed their effect on major adverse cardiovascular events, there has been limited assessment of their effectiveness on vascular aging in the coronary arteries specifically. However, the proliferation of CTCA as a common non-invasive imaging modality and the addition of FAI for post imaging analysis provides opportunities to measure the subclinical vascular aging response to therapy in the coronary arteries. Formalising this imaging modality as a method to measure and define phenotypes of normal and abnormal coronary arterial aging will help to better allocate pharmacotherapy to prevent or manage vascular aging, similar to atherosclerotic coronary artery disease (243).

### 5.1. Non-pharmacological management

Multiple studies have assessed non-pharmacological methods of reducing arterial stiffness, mainly looking at the effects of exercise, caloric restriction and a reduction in salt consumption (15). Habitual aerobic exercise has been postulated to protect the vasculature from age associated changes by increasing nitrous oxide availability in response to increased laminar shear stress (244), improving mitochondrial biogenesis and anti-oxidant enzyme production (245), and stimulating both the production and release of endothelial progenitor cells from the bone marrow (246, 247). A recent meta-analysis demonstrated that aerobic exercise of greater than 4 weeks significantly improved central arterial PWV (pooled mean difference [PMD]: −0.47 m/s [95% CI −0.68 to −0.25]), and this is supported by sub-group analysis of a further meta-analysis, which found the same for aerobic exercise in healthy (weighted mean difference [WMD]: −0.41 m/s [95% CI −0.55 to −0.27]) and hypertensive adults [weighted mean difference: −0.66 m/s (95% CI −1.23 to −0.10)] (248, 249).

The evidence of the effects of resistance or anaerobic exercise on arterial stiffness is more unclear. One meta-analysis of resistance exercise found it made no difference to PWV [WMD: −0.04 m/s (95% CI −0.42 to 0.34)], and this was consistent after sub-group analysis by participant and study characteristics (249). However, another meta-analysis found that resistance exercise increased cf-PWV [WMD 0.42 m/s (95% CI: 0.17–0.66)] (250). The potential negative effect of acute resistance exercise has been postulated to be due to greater eccentric muscle damage causing secretion of inflammatory products (250) and angiotensin II (251), which mediate endothelial cell signaling and dilatation. Another study suggested that intense resistance exercise may increase sympathetic nervous system activity and therefore chronically increase adrenergic vasoconstrictor tone and arterial stiffness (252).

Regarding dietary sodium, a meta-analysis of eleven studies demonstrated that an average daily salt reduction of 5.2 g results in a PMD of −2.8% (95% CI −5.08 to −0.51) in cfPWV (253). Caloric restriction has also been investigated as a non-pharmacological intervention to ameliorate vascular aging, given the robust evidence that it extends lifespan in multiple animal models (254). Caloric restriction in mouse models has been demonstrated to enhance nitrous oxide bioavailability, prevent age-induced endothelial dysfunction (241), and reduce oxidative stress (255). Short term weight loss based on caloric restriction in overweight and obese adults has been demonstrated to reduce cfPWV (−187 ± 29 cm/s in intervention group vs. 15 ± 42 cm/s in control group), however it is difficult to assess to what degree the effect is mediated by weight loss as opposed to the dietary change itself (256–258). However, there has been limited research into longer-term caloric restriction due to adherence issues and potential adverse effects (259). A 2 year RCT of caloric restriction in humans demonstrated a decrease in F2-isoprostanes which are an index of accumulated oxidative injury (260, 261). However, this study did not include a direct measure of vascular aging. Further characterization of the beneficial impacts of these non-pharmacological interventions on coronary arterial aging may help both identification of resilient and susceptible individuals based on their lifestyle habits and exploration of novel therapeutic targets by identifying the pathways through which these interventions mediate the pathophysiological process of vascular aging.

### 5.2. Anti-hypertensive agents

Much of the literature to date has focused on the efficacy of different classes of anti-hypertensives in attenuating vascular aging (262), including angiotensin converting enzyme (ACE) inhibitors, angiotensin receptor blockers, beta blockers, calcium channel blockers, nitrates and diuretics and aldosterone antagonists. Whilst there is evidence suggesting that all the classes can reduce arterial stiffening as measured by PWV, ACE inhibitors which mediate the renin-angiotensin-aldosterone-system (RAAS) have been suggested to be the most effective given that they change the intrinsic properties of the arterial wall (262–266). A recent meta-analysis has also demonstrated that this effect from ACE inhibitors [PMD: −1.69 m/s (95% CI −2.05 to −1.33)] is independent of blood pressure reduction (263), through a direct influence on arterial wall structure, a reduction in oxidative stress and mediation of the pro-inflammatory state elucidated above (267). Further evidence is also emerging about the benefits of RAAS blockade in slowing aging via downregulation of mechanistic target of rapamycin and modulation of sirtuin expression (268).

### 5.3. Lipid-lowering agents

Statins have been demonstrated to have a benefit in preventing CVD events in moderate risk populations or those with a history of CVD [pooled RR: 0.72 (95% CI 0.64–0.81)] (269). This effect is primarily through a reduction in the amount of circulating low density lipoprotein (LDL), however there is also evidence that statins mediate inflammation independently of this as well (270). PCSK9 inhibitors can further improve outcomes in patients [pooled RR: 0.83 (95% CI 0.78–0.88)] already treated with standard therapy via further reduction of circulating LDL (271, 272). In patients who have signs of coronary arterial aging in the form of atherosclerosis as measured by CTCA, statins have been demonstrated to reduce major adverse cardiovascular events adjusted for traditional risk factors, age, sex and atrial fibrillation [HR: 0.76, (95% CI 0.60–0.95)], with the benefit being related to the degree of CAC (273). Numerous trials have also demonstrated that statins also confer a reduction in cfPWV in obese middle aged and older adults (treatment group: −163 ± 21 cm/s vs. control group: −48 ± 34 cm/s), independent of any changes in blood pressure (259, 274).

### 5.4. Diabetic agents

Whilst the evidence around the effect of glucose lowering on vascular outcomes in patients with type 2 diabetes is mixed (275, 276), certain classes of diabetic agents including metformin, GLP-1 agonists and DPP-4 inhibitors, have been demonstrated to improve mediate vascular aging through improving endothelial function and arterial stiffness, independent of glucose control (277–279). A recent meta-analysis demonstrated that SGLT-2 inhibitors significantly improve endothelial function as measured by FMD [PMD: 0.95% (95% CI 0.18–1.73)], and DPP-4 inhibitors [PMD: −0.18 m/s (95% CI −0.3 to −0.07)], SGLT-2 inhibitors [PMD: −1.30 m/s (95% CI −2.41 to −0.19)] and GLP-1 receptor agonists [PMD: −1.97 m/s (95% CI −2.65 to −1.30)] significantly decrease arterial stiffness in type 2 diabetic patients as measured by PWV (242, 278). There has been limited exploration of these novel diabetic agents in patients demonstrating coronary arterial aging and subclinical coronary atherosclerosis, however a pooled cohort analysis demonstrated that the effect modification of GLP-1 agonists on major adverse cardiovascular events was mediated by the degree of subclinical atherosclerosis (280).

### 5.5. Colchicine

Colchicine is a drug derived from the *Colchicum Autumnale* plant with notable anti-inflammatory effects through the inhibition of tubulin polymerization and microtubule generation (281). It is routinely used for the treatment of gout, familial Mediterranean fever and pericarditis (282). Given the significant role of inflammation in the development of atherosclerosis, the use of colchicine in the prevention of CVD events has been studied in three large RCTs. The LoDoCo and LoDoCo2 trials looked at the efficacy of colchicine in preventing major adverse cardiovascular events in patients with stable CAD (283, 284). Both the LoDoCo [HR: 0.33 (95% CI: 0.18–0.59); *p* < 0.0001] and LoDoCo2 [HR: 0.69 (95% CI: 0.57–0.83); *P* < 0.001] trial demonstrated that 0.5 mg of colchicine daily significantly decreased the risk of coronary events (285). The COLCOT trial explored if the impact of commencing colchicine in patients with a myocardial infarction on cardiovascular events. It found that there was a significant reduction in cardiovascular events over a median of 22.6 months [HR: 0.77 (95% CI: 0.61–0.96); *p* = 0.02] in patients who were commenced on 0.5 mg of colchicine daily within thirty days of their myocardial infarction (286).

There have also been small RCTs exploring the impact of colchicine on high sensitivity c-reactive protein (hs-CRP) in patients after an acute coronary syndrome, with mixed results. One study of 80 patients looked at the impact of giving patients 1 mg of colchicine daily to patients post acute coronary syndrome or stroke for thirty days and found no difference in hs-CRP (*p* = 0.22). However, another study of 80 patients looked at the impact of 0.5 mg of colchicine daily for 1 year and demonstrated a significant reduction in hs-CRP (treatment group: −37.3% vs. control group: −14.6%, *p* < 0.0001). To date, there have been no clinical trials looking at the effects of colchicine on PCAT inflammation, however this may change as CT FAI becomes more widespread.

### 5.6. Novel agents

The increasing focus on unravelling the mechanisms which drive vascular aging has resulted in testing of a range of novel therapeutic targets. These include senolytics, micro-RNA therapy, sirtuin activators, mTOR inhibitors, PPAR-gamma activators, antifibrotic agents and anti-inflammatory cytokine therapies (259, 266). Whilst many of these agents have clear therapeutic targets which are implicated in vascular aging, this has yet to be translated into clinical studies in human populations (259). mTOR inhibitors such as sirolimus and anti-proinflammatory cytokine therapies including tumor necrosis factor-alpha antagonists have demonstrated reductions in cfPWV in specific disease populations in which they are currently used (287–290). The CANTOS study looking at the effect of the IL-1β monoclonal antibody canakinumab also showed a reduction in major cardiovascular events with a three monthly 150 mg dose in patients with previous myocardial infarction [HR: 0.85 (95% CI 0.74–0.98)], providing support for further investigation of anti-proinflammatory therapies in patients with coronary arterial aging (291). Modulators of the sirtuin pathway including reservatrol and nicotinamide have shown some promise in animal models (292, 293), but clinical studies in human populations are scant (294). Similarly, senolytic and micro-RNA therapies have yet to be translated across to clinical trials in human populations, although a recent mouse model of senolytic therapy demonstrated senescent cells as a driver of the pro-inflammatory milieu, and a reduction in plaque growth and maladaptive plaque remodelling associated with treatment through transgenic and pharmacological approaches (295). Conversely, another recent study demonstrated that genetic senolysis of VSMCs did not change plaque size or composition, and pharmacological senolysis reduced atherogenesis in culture and *in vivo*, and senescent VSMCs in culture alone (296). There is currently no evidence regarding the effect of sirtuin modulators or senolytics on coronary arterial aging specifically, and thus it is critical to have a clearly defined biomarker of vascular aging for the coronary arteries to facilitate further study in this area Table 2.

**Table 2:** Comparison of treatment effects on markers of vascular aging.

Intervention	Trial	Type of study	Effect size	95% CI	*P*-value
Effect on FMD					
SGLT-2 (in patients with T2DM)	Batzias et al. (242)	Meta-analysis	PMD: 0.95%	0.18–1.73	*p* = 0.016
Effect on CVD events					
Statin (patients with 1 + traditional risk factor)	USPSTF (269)	Meta-analysis	PRR: 0.72	0.64–0.81	–
Statin (on CAC positive patients)	Mitchell et al. (273)	RCT	HR: 0.76	0.60–0.95	*p* = 0.015
Canakinumab 150 mg (in patients with previous MI)	Ridker et al. (291)	RCT	HR: 0.85	0.74–0.98	*p* = 0.021
PCSK9 inhibitor (vs. standard therapy)	Turgeon et al. (272)	Meta-analysis	PRR: 0.83	0.78–0.88	–
Colchicine (stable CAD patients)	Nidorf et al. (284)	RCT	HR: 0.33	0.18–0.59	*p* < 0.0001
Colchicine (stable CAD patients)	Nidorf et al. (285)	RCT	HR: 0.69	0.57–0.83	*p* < 0.001
Colchicine (in patients with MI in last 30 days)	Tardif et al. (286)	RCT	HR: 0.77	0.61–0.96	*p* = 0.02
Effect on central PWV					
Aerobic exercise	Huang et al. (248)	Meta-analysis	PMD:−0.47 m/s	−0.68 to −0.25 m/s	*p* < 0.0001
Aerobic exercise	Ashor et al. (249)	Meta-analysis	WMD: −0.39 m/s	−0.52 to −0.27 m/s	–
Resistance exercise	Ashor et al. (249)	Meta-analysis	WMD: −0.04 m/s	−0.42–0.34 m/s	–
Resistance exercise	Pierce et al. (250)	Meta-analysis	WMD: 0.42 m/s	0.17–0.66 m/s	*p* = 0.0008
Reduction in dietary sodium (by avg. 5.2 g/day)	D’elia et al. (253)	Meta-analysis	PMD: −2.84%	−5.08 to −0.51%	–
Caloric restriction (in overweight and obese middle aged and older adults)	Dengo et al. (258)	RCT	Intervention: −1.87 m/s Control: 0.15 m/s	Intervention: +/−0.29 m/s Control: +/−0.42 m/s	*p* < 0.05
ACE inhibitors (vs. placebo)	Shahin et al. (263)	Meta-analysis	PMD: −1.69 m/s	−2.05 to −1.33 m/s	*p* < 0.00001
Spironolactone (in hypertensive patients)	Mahmud et al. (297)	RCT	−1.54 m/s	+/- 0.2 m/s	*p* < 0.05
Atorvastatin (in overweight and obese middle-aged and older adults	Orr et al. (274)	RCT	Intervention: −1.63 m/s Control: −0.48 m/s	Intervention: +/- 0.21 m/s Control: +/- 0.34 m/s	*p* < 0.001
SGLT−2 inhibitors (in patients with T2DM)	Solini et al. (278)	RCT	-1.30 m/s	−2.41 to −0.19 m/s	*p* < 0.05
DPP-4 inhibitors (in patients with T2DM)	Batzias et al. (242)	Meta-analysis	−0.18 m/s	−0.30 to −0.07 m/s	*p* = 0.002
GLP-1 agonists (in patients with T2DM)	Batzias et al. (242)	Meta-analysis	−1.97 m/s	−2.65 to −1.30 m/s	*p* < 0.001

FMD, flow mediated dilation; PMD, pooled mean difference; CVD, cardiovascular disease; USPSTF, United States preventative services task force; PRR, pooled relative risk; CAC, coronary artery calcium; RCT, randomised control trial; HR, hazard ratio; MI: myocardial infarction; PWV, pulse wave velocity; WMD, weighted mean difference; ACE, angiotensin converting enzyme; T2DM, type 2 diabetes mellitus.

## 6. Conclusion

The continuing demographic trend towards an aging population has driven a concerted effort to define the pathophysiological mechanisms by which aging changes the structure and function of the vasculature. Manifestations of these mechanisms including increased arterial stiffness, intimal thickening and the absence of atherosclerosis have started to be incorporated into definitions of phenotypes of early or healthy vascular aging. Currently, these definitions are not yet unified, but do not often involve any assessment of the coronary arteries as their location and size results in measurement limitations of processes of vascular aging.

Advancements in CTCA have enabled more detailed measurement of atherosclerosis and of localised coronary artery inflammation. Given its widespread availability and accessibility it is the optimal imaging modality for assessment of vascular aging in the coronaries in large epidemiological and cohort studies. Recognising these measurement modalities as a formal assessment of coronary arterial aging is essential in guiding therapeutic trials, as they provide the endpoints and criteria for how efficacy of therapy is assessed. Forming a consensus around the criteria to define healthy and early vascular aging phenotypes in the coronary arteries is the first step in a more detailed analysis of how age driven vascular changes mediate resilience and susceptibility specifically to CAD. It will provide a framework to identify the mechanisms and conditions where age-driven changes in the vasculature increase the risk of developing CAD as opposed to representing physiological aging. Furthermore, it will help to identify novel therapeutic targets to modulate this risk based on the observed phenotypes of resilience and susceptibility.

Defining the phenotypes of resilience and susceptibility to vascular aging in the coronary arteries will facilitate exploration of novel risk factors and blood-based biomarkers that define these cohorts. This is made possible by recent advances in high throughput multi-omic analysis platforms and increased access to a wide range of patient data through large cohort studies and healthy aging biobanks. Furthermore, developments in functional and anatomical phenotyping of coronary artery pathophysiology, and clearer measures of angiographically “normal” vessels make it feasible to design discovery efforts to unravel the missing mechanisms of resilience and susceptibility to arterial aging. These advances and developments in culmination will help to facilitate unbiased approaches to identifying new blood-based markers of resilience and susceptibility to CAD and coronary events.
